# Carotid wall echogenicity at baseline associates with accelerated vascular aging in a middle-aged population

**DOI:** 10.1007/s10554-022-02760-3

**Published:** 2023-01-21

**Authors:** Emma Nyman, Per Liv, Per Wester, Ulf Näslund, Christer Grönlund

**Affiliations:** 1 Department of Public Health and Clinical Medicine, Umeå University, Umeå, Sweden; 2 Department of Radiation Sciences, Radiation physics, Biomedical engineering, Umeå University, Umeå, Sweden

**Keywords:** Asymptomatic, Atherosclerosis, Carotid intima media, Ultrasound, Echogenicity

## Abstract

Ultrasonic echolucent carotid intima-media (IM) complex and accelerated progression of carotid intima mediathickness (cIMT) have both separately been shown to predict future cardiovascular events. The aim of this studywas to evaluate if the echogenicity of the IM-complex is associated with the 3-year progression of cIMT. B-modeultrasound images captured at baseline and 3-year follow-up in the ‘Visualization of asymptomatic atherosclerotic disease for optimum cardiovascular prevention’ (VIPVIZA) trial were included (n: 3154). The bilateral mean cIMT and IM-echogenicity by greyscale median (GSM) were measured in the common carotid artery. Associations between IM-GSM at baseline and the 3-year cIMT progression were investigated using linear regression models for the whole population and stratified by sex, age and VIPVIZA study group (intervention versus control). In addition, adjusted analyses for confounding factors were performed. Unadjusted analysis showed that decreased IM-GSM at baseline was associated with increased progression of cIMT (p < 0.001). Stratified by age, the association was significant among 40 (p < 0.001) and 60 years old (p < 0.001). The association was statistically significant in both sexes and on comparison of VIPVIZA study subgroups. Adjustments for confounding factors did not alter the estimated relationship between IM-GSM and cIMT progression. Echolucent carotid intima media at baseline associates with increased 3-year cIMT progression among an asymptomatic, middle-aged population. Echogenicity of the intima media may identify individuals at risk for accelerated vascular aging.

**Supplementary Information:**

The online version contains supplementary material available at 10.1007/s10554-022-02760-3.

## Introduction

Cardiovascular diseases (CVD) such as myocardial infarction and stroke is the major cause of death globally [1]. The risk of suffer from a CVD can be measured by several well-known risk factors such as hypertension, hyperlipidaemia and smoking [2] However, risk stratification by traditional risk factors lacks in specificity since the CVD burden is still high. In addition to traditional risk factors, measurements of unfavourable vascular aging could be useful in identification of individuals at risk of developing CVD. Unfavourable vascular aging includes functional changes such as stiffening and endothelial dysfunction but also structural changes with arterial wall thickening [3]. These changes in the arterial wall can lead to accelerated vascular aging, which may be measured by carotid intima media thickness [4, 5]. In a recently published meta-analysis it was shown that interventions which reduced progression of cIMT were associated with decreased cardiovascular risk [6]. This emphasises that identification accelerated progression of cIMT could be valuable and prevention of accelerated vascular aging may decrease the risk of CVD.

Assessment of the carotid intima media (IM) complex composition by its ultrasound-based echogenicity could be an additional parameter to identify individuals at risk for accelerated vascular aging. Previous studies evaluating the IM-echogenicity have repeatedly found that decreased echogenicity of the IM-complex associate with low high-density lipoprotein (HDL)-cholesterol levels and increased body mass index (BMI) [7–12]. Among an elderly population inflammation markers have further been associated with IM-echogenicity [7] and decreased echogenicity of the IM-complex has been shown to be associated with an increasing number of arterial territories affected by atherosclerosis [13]. In addition, cIMT has been found to be inversely related to IM-GSM [11]. There is evidence that the IM-echogenicity predicts CVD events independent of traditional risk factors [14], whereas in an elderly male population the IM-echogenicity was a predictor for both CVD and all cause death [15].

Results from previous studies suggest that the echogenicity of the IM-complex may provide information beyond the information gained from single cIMT assessment [8, 11]. To the best of our knowledge, the relationship of IM-complex echogenicity with cIMT progression has not been evaluated. We hypothesised that IM-complex with lower echogenicity is associated with an accelerated cIMT progression in an asymptomatic, middle age population. This study aimed to evaluate if the echogenicity (GSM) of the carotid IM-complex is associated with cIMT progression over a 3-year follow-up period in an asymptomatic, middle-aged population.

## Materials and methods

### Study population

VIPVIZA (Visualization of asymptomatic atherosclerotic disease for optimum cardiovascular prevention) is a prospective, population based randomized controlled trial (RCT) which has previously been described in detail [16, 17]. In brief, inhabitants in the county of Västerbotten, Sweden, are invited during the year they turn 40, 50 or 60 years to attend Västerbotten Intervention Program (VIP). VIP is a CVD prevention program integrated in the regular primary health care including CVD risk factor measurements, questionnaires regarding life style habits and individual counselling [18]. The VIPVIZA study originates from the routine prevention program and VIP participants aged 40 and 50-years old with one or more traditional risk factor for CVD, and 60-years old regardless of risk factor profile, were invited to participate in the VIPVIZA study. The inclusion criteria were set to target a population with low to intermediated risk of CVD. VIPVIZA participants underwent an ultrasound examination of the carotid arteries with the aim of measuring vascular age and subclinical atherosclerosis by cIMT and plaque identification. At baseline, the intervention group (n: 1749) and their primary care physicians received a pictorial presentation of the degree of subclinical atherosclerosis based on the ultrasound assessment. For each individual, the measured cIMT was compared to age and sex matched normal values and their relative vascular age was presented as a colored gauge (green or yellow illustrated vascular age lower than expected for your actual age, and orange or red for vascular age older than your actual age). Traffic lights illustrated plaque formation, showing red light for plaque presence and green light for plaque absence. The control group (n: 1783) and their physicians proceeded with the routine VIP prevention but did not obtain the pictorial presentation. At the 3-year follow-up the same examinations were performed again, including the carotid ultrasound scan.

### Carotid Ultrasound

**Fig. 1:**
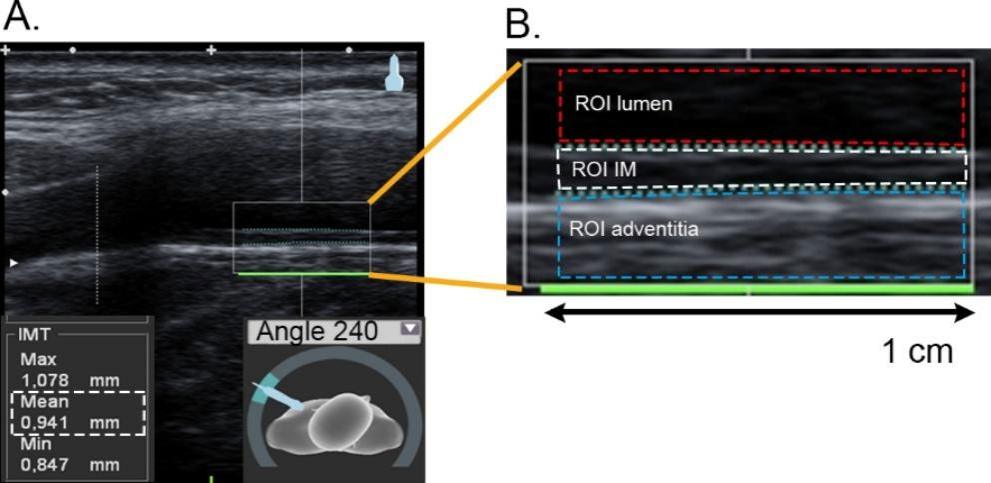
Carotid intima media thickness (cIMT) and intima media greyscale median (IM-GSM) measurements in the study. **(A)** Automatic real-time cIMT measurement in the distal 1-cm of the far wall of common carotid artery at predefined angle (240°). **(B)** Measurement of IM-GSM by automatic detection within square containing cIMT measure. Region of interest (ROI) automatic detected in lumen and adventitia for image normalisation. IM-complex automatic cropped by the boundaries used for cIMT measurements

The baseline and 3-year follow-up ultrasound examinations were performed using a portable ultrasound system Cardio Health Station (CHS) with a linear 7 MHz transducer (Panasonic Healthcare Corporation of North America, Newark, NJ, USA). The ultrasound examinations followed a standardised imaging protocol which has previously been described [16]. The CHS measures cIMT automatically in real-time from a 1-cm segment of distal far wall of the common carotid artery (CCA) in longitudinal projections (Fig. 1 A). The measurements were performed at predefined angles bilaterally.

In the present study, cIMT images measured at 240° (left mean cIMT) and 120° (right mean cIMT) both in the baseline and the 3-year follow-up were used for calculating the bilateral mean cIMT. The inter-operator variability regarding cIMT measures has previously been determined for our technique, where the intra class correlation coefficient was estimated to be as high as 0.95[19]. The CHS stores the ultrasound images as screen dump images in the jpg format, where the cIMT segment is included.

### Intima Media Echogenicity

The echogenicity of the IM-complex was quantified using the greyscale median (GSM) descriptor. The computation of the GSM followed a standard procedure [20] where inter-subject differences in absolute echogenicity are adjusted for by using a normalisation procedure (Fig. 1B). The intima-lumen and adventitia-media borders are overlaid on the B-mode image with green dotted lines in the stored CHS images. The IM-complex was automatically segmented as the greyscale image region within these borders (excluding the green dotted lines) (Fig. 1B).

A 0.25 × 1 cm region of interest (ROI) was automatically placed in the lumen, immediately above the upper border of the IM-complex, and the mean intensity was computed. Next, a 0.25 × 1 cm ROI was automatically placed immediately below the lower border of the IM complex, and the maximal intensity of the adventitia was computed. Based on these values, the greyscale of the image was linearly rescaled such that the lumen intensity was set as 0 (darkest) and the adventitia as 190 (brightest).

The IM-GSM was finally computed as the median of the pixel intensities of the IM-complex of the normalised image. The bilaterally mean IM-GSM was calculated. The computations were made using an in-house (Department of Biomedical engineering, Region Västerbotten, Umeå, Sweden) MATLAB software (2018b, MathWorks, Nattick, MA, USA).

### Statistics

Associations between IM-GSM and 3-year cIMT progression were investigated using linear regression, with cIMT at 3-year follow-up as the dependent variable while adjusting for baseline IM-GSM. The IM-GSM values were modelled using restricted cubic splines to allow for a possible non-linear effect. The splines were connected using three knots placed at the 10th, 50th and 90th percentiles of the IM-GSM distribution in the study population. Mean values calculated from both the left and the right side were used in all analyses. The cIMT-variable (both baseline and 3-year follow-up) was transformed using the natural logarithm to reduce skewness of residuals and to improve the fit of the statistical model. Predicted cIMT at 3-years on the logarithmic scale was calculated for different values of IM-GSM, while adjusting the model at the median baseline cIMT across all individuals on the logarithmic scale (= log (0.64 mm)). The predicted progression of cIMT was calculated by re-transforming the predicted 3-year cIMT from logscale into original unit (i.e., mm) using the exponential function, and thereafter subtracting the median baseline cIMT (= 0.64 mm). The results were presented in graphs displaying the cIMT-progression’s association to IM-GSM, accompanied by 95% confidence interval (CI) band. The model adjustment made at the median baseline cIMT implies that graphs illustrate cIMT-progress for a “typical” individual, other values of baseline cIMT would move the curve vertically but not alter the curve’s shape or slope.

Heterogeneity in the associations was investigated using subgroup analyses, using sex, age groups and VIPVIZA study group, respectively to stratify the regression analysis. Additionally, models with interactions between IM-GSM and sex, age groups and VIPVIZA study group, was fitted respectively, to formally test for heterogeneity in the associations found.

To explore possible causes of the association between IM-GSM and cIMT-progression, we fitted additional regression models which adjusted for possible joint causes of IM-GSM and cIMT-progression. The intention was to investigate whether the relationship could be altered by adjusting for the confounding factors which are possibly involved in generating the association. All models were adjusted for sex, age group and intervention group. Additionally, the models were adjusted for the following baseline variables: Hypertension diagnosis at baseline defined as 1, normal (systolic blood pressure < 140 mmHg, diastolic blood pressure < 90); 2, controlled hypertension; 3, uncontrolled hypertension; 4, hypertension without treatment. Lipid lowering medication at baseline 1, yes; 2, no. Low density lipoprotein (LDL) cholesterol / high density lipoprotein (HDL) cholesterol quota (mmol/l). Body mass index (BMI) and blood sugar fasting (mmol/l), respectively. An unadjusted regression model (not adjusted for sex, age group, intervention group or confounding factors) and a fully adjusted regression model adjusted for all mentioned confounding factors was also performed. The results of the adjusted linear model was also stratified with respect to age and sex.

Sensitivity analysis was carried out by re-running all regression analyses using multiple imputation of missing data, where the results were pooled across 10 imputed data sets. Imputations were made after log-transformation of cIMT variables, using the aRegImpute function from the R-package HMisc.

Regression analyses was performed in R version 4.1.1 using the rms package (R Core Team (2021). R: A language and environment for statistical computing. R Foundation for Statistical Computing, Vienna, Austria). Significance level was set at 0.05 for all tests.

The inter-operator variability of IM-GSM was assessed using IM-GSM estimates from two repeated ultrasound scans (back-to-back) by two separate sonographers from 27 study participants [21]. The operators were blinded from each other’s results. The inter-class correlation (ICC) metric was used to assess the inter-operator variability.

This study was approved by the Umeå Regional Ethical Review Board (Dnr 2011-445-31 M, 2012-463-32 M, 2013_373 − 32 M), and all participants provided written informed consent.

## Results

3154 (89.3%) of the 3532 participants recruited to the baseline VIPVIZA completed the 3-year follow-up ultrasound examination, which constitutes the present study’s population. Of the 378 participants that had dropped out at the 3-year follow-up 207 had been lost to follow-up, 114 had withdrawn consent, 37 had moved out of the region and 20 had died.

The baseline characteristics of the 3154 VIPVIZA participants are presented in Table 1. The majority of the study participants were 60-years at baseline (65.5%) and half of the study population was female (53.5%). At baseline mean cIMT was 0.663 (± 0.145) mm, and mean IM-GSM was 21.3 (± 10.2) in the total population. Mean cIMT was 0.615 among 40-years old and increased to 0.773 among 60-years old. Further, mean IM-GSM was 26.1 (± 10.7) among 40 years-old and 20.6 (± 9.0) among 60-years old.

The inter-observer variability of the IM-GSM measurements was found to be 0.75 (ICC).

**Table 1:** Baseline characteristics (n: 3154)

	Total	40-years	50-years	60-years	Female	Male
40 years	233 (7.4%)	-	-	-	121 (7.2%)	112 (7.6%)
50 years	856 (27.1%)	-	-	-	450 (26.7%	406 (27.7%)
60 years	2065 (65.5%)	-	-	-	1117 (66.2%)	948 (64.7%)
Female	1688 (53.5%)	121 (51.9%)	450 (52.6%)	1117 (54.1%)	-	-
Systolic blood pressure (mmHg)	129.2 (± 16.0)	121.5 (± 13.7)	129.1 (16.2)	130.1 (15.9)	126.9 (± 16.2)	131.8 (15.4)
Diastolic blood pressure (mmHg)	82.5 (± 10.3)	78.8 (± 10.6)	83.9 (± 11.1)	82.3 (± 9.7)	80.7 (± 9.9)	84.6 (10.3)
Total cholesterol (mmol/l)	5.60 (± 1.08)	5.02 (± 1.03)	5.64 (± 1.04)	5.67 (± 1.08)	5.69 (± 1.04)	5.50 (1.12)
Triglycerides (mmol/l)	1.47 (± 0.95)	1.34 (± 1.47)	1.59 (± 1.08)	1.43 (± 0.79)	1.31 (± 0.71)	1.65 (1.13)
High density lipoprotein cholesterol (mmol/l)	1.39 (± 0.42)	1.29 (± 0.37)	1.31 (± 0.39)	1.43 (± 0.43)	1.53 (± 0.43)	1.22 (0.34)
Low density lipoprotein cholesterol (mmol/l)	3.55 (± 0.97)	3.11(± 0.82)	3.61 (± 0.94)	3.58 (± 0.99)	3.56 (± 0.95)	3.54 (1.00)
Current smoker	372 (11.8%)	25 (10.7%)	113 (13.2%)	234 (± 11.4%)	203 (12.1%)	169 (11.5%)
Family history of cardiovascular disease	1011 (32.0%)	233 (100%)	304 (35.6%)	474 (23.0%)	559 (33.3%)	413 (28.2%)
Diabetes	206 (6.6%)	6 (2.6%)	50 (5.8%)	150 (7.3%)	89 (5.4%)	117 (8.0%)
Body mass index	27.6 (± 4.7)	27.2 (± 5.1)	28.7 (± 5.0)	27.2 (± 4.6)	27.32 (± 5.2)	27.93 (4.2)
SCORE^a^	1.3 (1.2)	0.2 (± 0.2)	0.6 (± 0.5)	1.7 (± 1.3)	0.69 (± 0.49)	1.94 (1.37)
Framingham risk score^b^	12.8 (9.3)	3.9 (± 2.6)	9.9 (± 6.7)	14.9 (± 9.7)	8.25 (± 5.37)	17.94 (10.1)
Mean cIMT^c^ (mm)	0.663 (± 0.145)	0.615 (± 0.098)	0.692 (± 0.130)	0.773 (± 0.161)	0.709 (± 0.137)	0.775 (0.172)
Mean IM-GSM^d^	21.3 (± 10.2)	26.1 (± 10.7)	21.9 (± 9.7)	20.6 (± 9.0)	21.6 (± 9.8)	21.2 (± 9.0)

mean (± SD) or n (%)

^a^Systematic coronary risk evaluation, absolute 10-year risk in %

^b^Absolute 10-year risk in %

^c^Carotid intima media thickness

^d^Intima-media greyscale median

### Unadjusted model, total VIPVIZA population

**Fig. 2:**
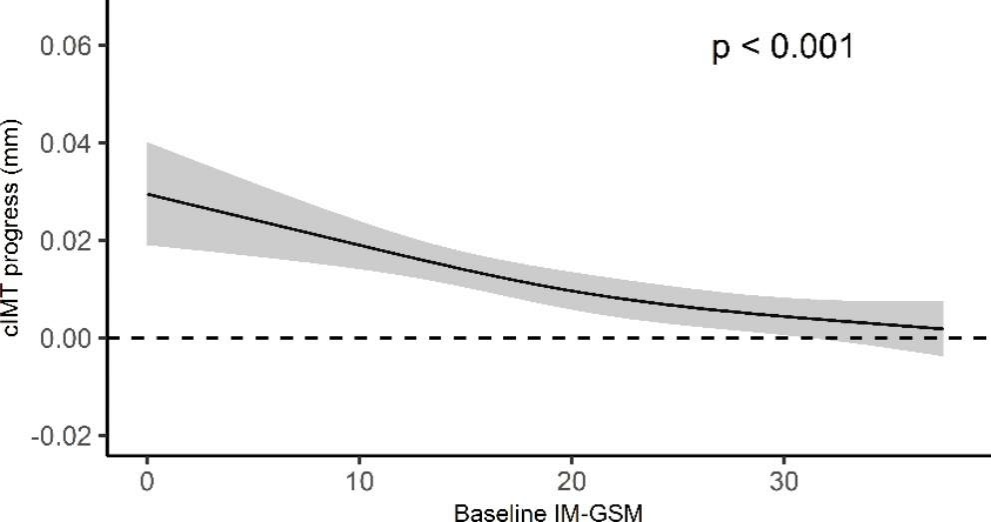
Estimated association between baseline intima media greyscale median (IM-GSM) and 3-year carotid intima media thickness (cIMT) progression from unadjusted regression models for total study population (n: 3154). Solid line shows expected value of cIMT progress, and the grey band represents its 95% confidence interval. Dotted line at a 3-year cIMT progression of zero

There was a significant association between baseline IM-GSM and the progression of cIMT in the 3-year follow-up period where the progression was highest among individuals with lower baseline GSM values (p < 0.001) (Fig. 2).

### Subgroup analyses and adjusted model

**Fig. 3:**
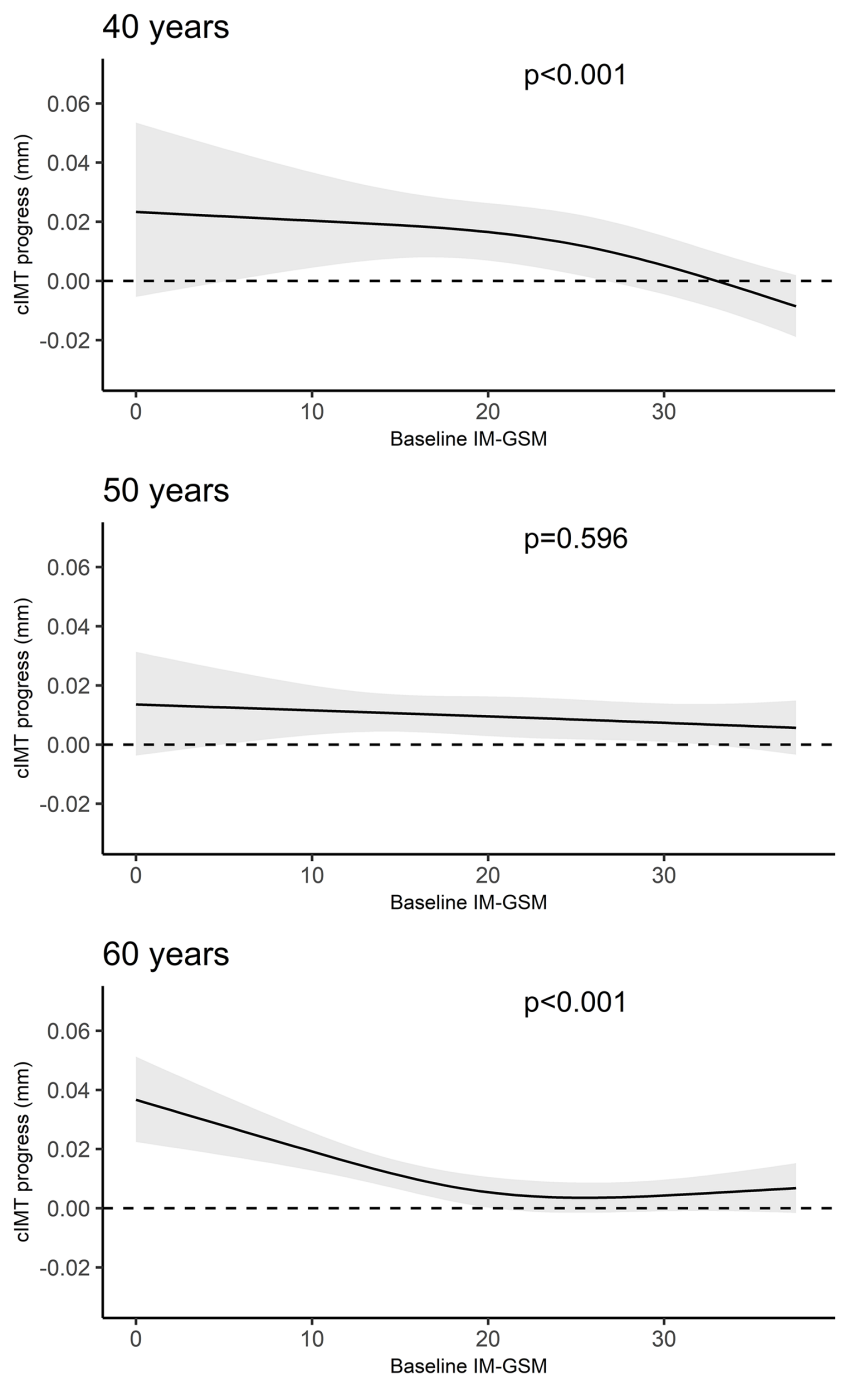
Estimated association between baseline intima media greyscale median (IM-GSM) and 3-year carotid intima media thickness (cIMT) progression based on age subgroups. Solid line shows expected value of cIMT progress, and the grey band represents its 955% confidence interval. Dotted line at a 3-year cIMT progression of zero

The association between baseline IM-GSM and cIMT progression was present amongst 40 (p < 0.001) and 60 years sold (p < 0.001), however no associations could be found among 50 years old (p = 0.596) (Fig. 3). The interaction between baseline GSM and age group was statistically significant (p = 0.001).

**Fig. 4:**
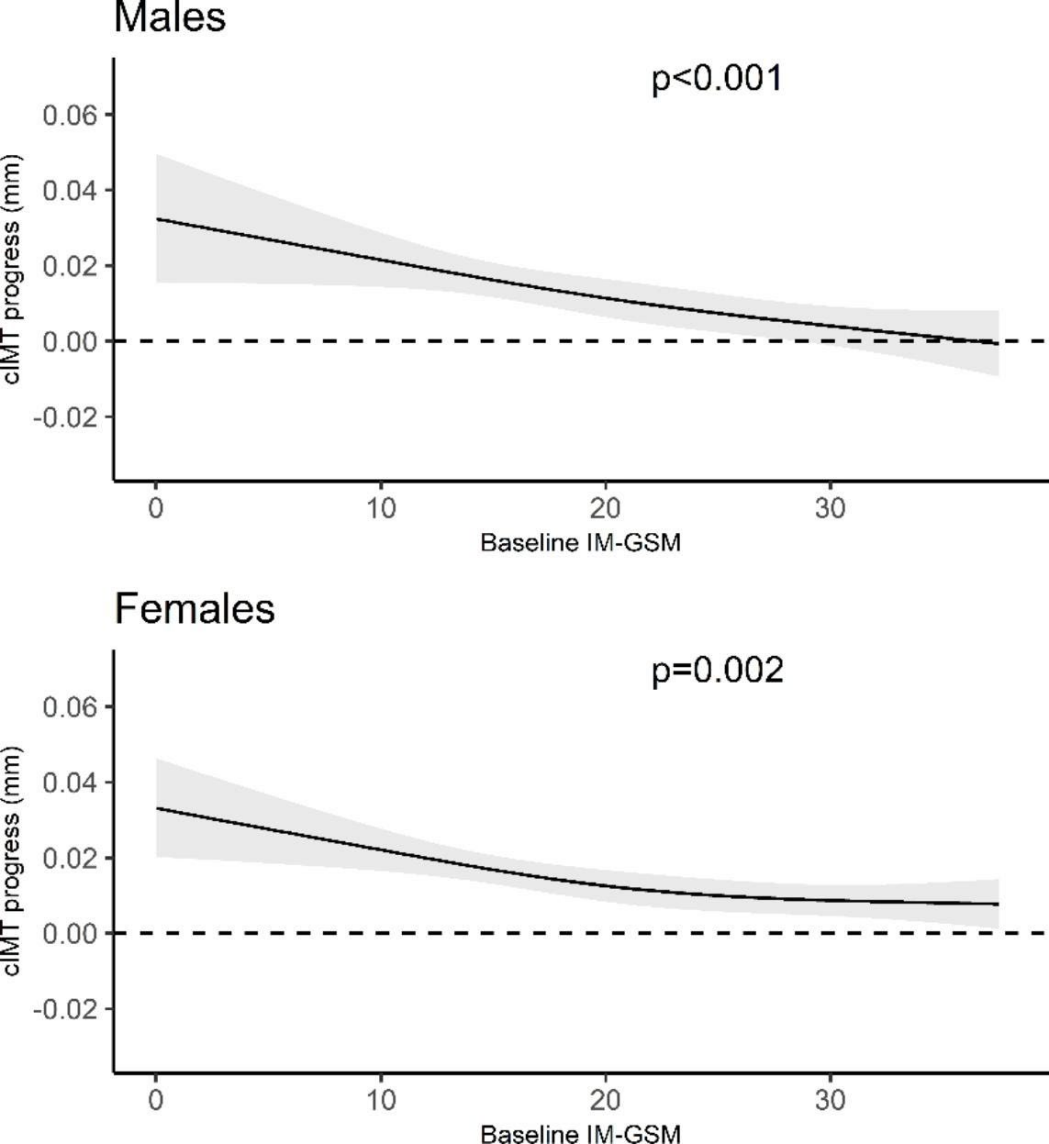
Estimated relationship between baseline intima media greyscale median (IM-GSM) and 3-year carotid intima media thickness (cIMT) progression based on male and female subgroups. Solid line shows expected value of cIMT progress, and the grey band represents its 95% confidence interval. Dotted line shows 3-year cIMT progression of zero

The association between baseline IM-GSM and cIMT progression was statistically significant for both the male and female subgroups (Fig. 4).

Analyses stratified by study group (intervention versus control) showed similar association between IM-GSM and cIMT progression in the two groups (results not shown). Furthermore, we statistically tested a possible interaction between IM-GSM and study group, to examine if study group interfered with the relationship between IM-GSM and cIMT progression. The interaction was found to be not statistically significant (p = 0.75).

Adjustments for possible confounder factors did not alter the estimated relationship between IM-GSM and cIMT progression significantly in the total population (Table 2).

**Table 2:** Regression coefficient () with 95% confidence interval (CI) for the effect of intima media greyscale median (IM-GSM) on carotid intima media thickness (cIMT) progression for an unadjusted regression model and for models adjusted for corresponding confounding factors, and for a fully adjusted model. p-value for test of IM-GSM and cIMT progression relationship. Note that the use of non-linear terms using splines implies that the slope is not constant for all values of IM-GSM. Here we display the for the median of IM-GSM.

βββ		
Confounding factor	β mm (95% CI)	p
Unadjusted model	-0.014 [-0.019, -0.009]	< 0.001
Hypertension diagnosis	-0.012 [-0.018, -0.007]	< 0.001
LDL/HDL^*^ quota	-0.012 [-0.017, -0.006]	< 0.001
Lipid lowering medication	-0.012 [-0.017, -0.006]	< 0.001
Body mass index	-0.013 [-0.019, -0.008]	< 0.001
Fasting blood sugar	-0.012 [-0.017, -0.006]	< 0.001
Fully adjusted model	-0.013 [-0.018, -0.007]	< 0.001

^*^Quota low density lipoprotein (LDL) cholesterol / high density lipoprotein (HDL) cholesterol

The age- and sex-stratified analyses showed similar patterns as when performed on the full dataset; adjusting for the covariates did not affect the relationship between IM-GSM and cIMT to a substantial degree (Supplementary Table 1).

The analysis employing multiple imputation gave similar results (results not shown). In total 378 participants did not participate in the 3-year follow-up ultrasound examination. Additional, 3 participants in the 3-year follow-up did not have cIMT measured in the right carotid artery due to poor image quality. Missing values of cIMT at 3 years follow-up: left n: 378 and right side n: 381.

## Discussion

This study shows that echolucent carotid IM-complex at baseline associated with accelerated progression of cIMT during a 3-year follow-up period in an asymptomatic, middle-aged population with low to moderate risk of CVD. Further this association remained when adjusting for possible confounding factors. To the best of our knowledge, this is the first study showing an association of carotid IM echogenicity with cIMT progression.

The echogenicity of the IM-complex has been suggested to add additional information beyond the information that can be gained from cIMT measurements [11–14]. This hypothesis is supported by the results of this study. Echogenicity of the carotid IM-complex could be a valuable tool for identification of individuals with accelerated vascular aging defined by progressive cIMT. Since increased progression of cIMT has been shown to be associated with increased CVD risk [6], identification of progression-prone arterial walls could offer new information. With increasing age the echogenicity of the intima media layer has been demonstrated to decrease [22] and the arterial wall thickness increase [23].

In our study the association of IM-GSM to progression of cIMT differed between age groups. The association was statistically significant among 40 and 60 years old, but not among the 50 years old. The 40 years old only accounted for 7.4% of the total study population, which limits the generalisability of the results in this age group. The 60-years age group constitutes 65% of the study population and was included due to age only. For participants 40 or 50-years old, the inclusion depended on both age and possession of traditional risk factors. The difference in inclusion criteria for the different age groups in the VIPVIZA study could possibly contribute to the differences between age-groups.

Importantly, the association of IM-GSM and cIMT progression found in our study was similar in both sexes. It is well known that among a middle-aged population males have thicker cIMT than females and have a higher prevalence of carotid plaques [24]. Since sex differences at the arterial wall level is present, it’s important to identify strategies that contributes to both sexes.

The association was still present after adjusting for traditional risk factors, which indicates that the causal mechanism behind the association is complex and includes additional factors, not investigated in the present study. In a male population, IM-echogenicity was related to obesity and insulin resistance independent of cIMT, suggesting that IM-echogenicity could act as a potential marker of unfavourable metabolic function [10]. This has previously been observed in young subjects which showed that IM-GSM was mainly related to BMI, whereas cIMT was more tightly associated with the cardiovascular risk factors of age and blood pressure [8]. Although prior studies have found associations between IM-GSM and risk factors, our results emphasise that the relationship and interaction between risk factors, IM-GSM and cIMT progression is multifaceted.

In the Prospective Investigation of the Vasculature in Uppsala Seniors (PIVUS) study, comprised of an elderly population who were examined with ultrasound to measure IM-echogenicity and cIMT, the authors demonstrated that dyslipidaemia, oxidative stress and inflammation variables were related to IM-echogenicity [7]. Furthermore, low shear stress was associated with a thick and an IM-complex with decreased echogenicity [25]. In our study, no variables reflecting inflammation or stress were available for analysis, thus we were unable to evaluate its influence on the associations found in this study.

The echogenicity of the IM-complex has the potential to act as a marker for accelerated progression of cIMT which indicates accelerated vascular aging. It may serve as a tool for early detection and screening for individuals needing preventive treatment beyond single measurement of cIMT.

### Limitations

First, IM-GSM was evaluated in terms of cIMT progression and not hard endpoints. The usefulness of IM-GSM in this population to predict CVD events remains to be explored. Further, the causal mechanism of the association between IM-GSM and cIMT needs to be further explored. The adjusted analysis in this study did not include variables reflecting inflammation which possible could alter the association. Further analysis of additional factors, such as biomarkers contribution will be of great interest in this population.

## Conclusion

Decreased echogenicity of carotid intima media complex at baseline was associated with 3-year progression of cIMT in an asymptomatic, middle-age population with low to intermediate risk for CVD. Echogenicity of the carotid intima media may be one way to identify accelerated vascular aging defined as accelerated progression of cIMT and act as a valuable tool in future CVD prevention.

## Electronic supplementary material

Below is the link to the electronic supplementary material.

Supplementary Material 1

## Declarations

### Statements and declarations

Founding.

This work was supported by Region Västerbotten (Central ALF, Dnr ALFVLL-298001 and ALFVLL643391), the Swedish Research Council (Dnr 521-2013-2708, 2016 − 01891), the Heart and Lung Foundation (Dnr 20150369, 20170481), SKANDIA Risk & Health, and an unconditional donation from Carl Bennet Ltd, Sweden. VIPVIZA was also funded by the Swedish Society of Medicine, the Heart Foundation in Northern Sweden, STROKE – the national association, The Swedish Insurance Society, Visare Norr (the four Northern Regions), and the Swedish and the Västerbotten Heart and Lung Associations. The funders of the study had no role in the study design, data collection, data interpretation, or writing of the report.

Author Contributions.

All authors contributed to the study conception and design. Material preparation, data collection and analysis were performed by Emma Nyman, Per Liv and Christer Grönlund. The first draft of the manuscript was written by Emma Nyman and all authors commented on previous versions and approved the final manuscript.
