# Supplementary material for: Carotid wall echogenicity at baseline associates with accelerated vascular aging in a middle-aged population
Source: Int J Cardiovasc Imaging. 2023 Jan 21;39(3):575–83. doi: 10.1007/s10554-022-02760-3 (PMC9947053; doi:10.1007/s10554-022-02760-3)
Supplement: Supplementary file 1 — Supplementary Material 1 [file 10554_2022_2760_MOESM1_ESM.docx]

| **Supplementary Table 1.** Adjusted analyses stratified by age-group and sex. Regression coefficient (β) with 95 % confidence interval (CI) for the effect of intima media greyscale median (IM-GSM) on carotid intima media thickness (cIMT) progression for an unadjusted regression model and for models adjusted for corresponding confounding factors, and for a fully adjusted model. p-value for test of IM-GSM and cIMT progression relationship. Note that the use of non-linear terms using splines implies that the slope β is not constant for all values of IM-GSM. Here we display the β for the median of IM-GSM.  **Sex – Males** | | |
| --- | --- | --- |
| Confounding factor | β mm (95 % CI) | p |
| Unadjusted model | -0.016[-0.024, -0.007] | < 0.001 |
| Hypertension diagnosis | -0.014 [-0.023, -0.006] | 0.002 |
| LDL/HDL^*^ quota | -0.015 [-0.023, -0.006] | 0.002 |
| Lipid lowering medication | -0.015 [-0.024, -0.007] | 0.002 |
| Body mass index | -0.014 [-0.022, -0.005] | 0.004 |
| Fasting blood sugar | -0.014 [-0.022, -0.005] | 0.006 |
| Fully adjusted model | -0.015 [-0.024, -0.006] | 0.004 |
| **Sex – Females** |  |  |
| Confounding factor | β mm (95 % CI) | p |
| Unadjusted model | -0.013[-0.020, -0.006] | 0.002 |
| Hypertension diagnosis | -0.011 [-0.018, -0.004] | 0.009 |
| LDL/HDL^*^ quota | -0.010 [-0.017, -0.003] | 0.022 |
| Lipid lowering medication | -0.011 [-0.018, -0.004] | 0.007 |
| Body mass index | -0.012 [-0.019, -0.004] | 0.006 |
| Fasting blood sugar | -0.010 [-0.017, -0.003] | 0.011 |
| Fully adjusted model | -0.011 [-0.019, -0.004] | 0.007 |
| **Age-group 40 years** |  |  |
| Confounding factor | β mm (95 % CI) | p |
| Unadjusted model | -0.014[-0.038, 0.005] | <0.001 |
| Hypertension diagnosis | -0.018 [-0.039, 0.003] | <0.001 |
| LDL/HDL^*^ quota | -0.016 [-0.038, 0.006] | <0.001 |
| Lipid lowering medication | -0.016 [-0.038, 0.006] | <0.001 |
| Body mass index | -0.018 [-0.041, 0.004] | 0.001 |
| Fasting blood sugar | -0.015 [-0.037, 0.006] | 0.011 |
| Fully adjusted model | -0.022 [-0.045, 0.002] | 0.007 |
| **Age-group 50 years** |  |  |
| Confounding factor | β mm (95 % CI) | p |
| Unadjusted model | -0.004[-0.014, 0.005] | 0.596 |
| Hypertension diagnosis | -0.004 [-0.014, 0.005] | 0.561 |
| LDL/HDL^*^ quota | -0.004 [-0.014, 0.006] | 0.563 |
| Lipid lowering medication | -0.006 [-0.016, 0.005] | 0.424 |
| Body mass index | -0.005 [-0.015, 0.005] | 0.617 |
| Fasting blood sugar | -0.004 [-0.014, 0.005] | 0.605 |
| Fully adjusted model | -0.006 [-0.016, 0.005] | 0.424 |
| **Age-group 60 years** |  |  |
| Confounding factor | β mm (95 % CI) | p |
| Unadjusted model | -0.012[-0.019, -0.006] | <0.001 |
| Hypertension diagnosis | -0.013 [-0.020, -0.006] | <0.001 |
| LDL/HDL^*^ quota | -0.012 [-0.019, -0.005] | <0.001 |
| Lipid lowering medication | -0.012 [-0.019, -0.005] | <0.001 |
| Body mass index | -0.014 [-0.021, -0.007] | <0.001 |
| Fasting blood sugar | -0.012 [-0.019, -0.005] | <0.001 |
| Fully adjusted model | -0.0013 [-0.020, -0.006] | <0.001 |
| ^*^Quota low density lipoprotein (LDL) cholesterol / high density lipoprotein (HDL) cholesterol. | | |
